# The importance of rapid aneuploidy screening and prenatal diagnosis in the detection of numerical chromosomal abnormalities

**DOI:** 10.1186/2193-1801-2-490

**Published:** 2013-09-29

**Authors:** Ghada M Elsayed, Lobna El Assiouty, Ezzat S El Sobky

**Affiliations:** Clinical pathology Department, National cancer institute, Cairo University, Giza, Egypt; Genetic consultant, National screening program for Women and Child Health, Abu Dhabi, United Arab Emirates; Genetics Unit, Pediatrics Department, Ain Shams University, Cairo, Egypt; Medical genetics center, 27A Baghdad Street, Korba Cairo, Egypt

**Keywords:** Prenatal diagnosis, Amniocentesis, Chromosomal anomalies, Down syndrome, FISH

## Abstract

**Objectives:**

Evaluation of Fluorescent in situ hybridization (FISH) as a tool for rapid aneuploidy screening (RAS) of high risk pregnancies, before its approval in the national antenatal screening and genetic diagnosis program in Egypt.

**Methods:**

The cytogenetic data of prenatal specimens, and results of FISH of 100 patients performed between, January 2009 and December 2009, at the Medical Genetics Center (MGC) laboratory were retrieved and reviewed. AneuVysion Assay kit was used for detection of 13, 21, X, Y, 18 aneuploidies.

**Results:**

Maternal age varied from 21 to 44 years (mean was 35.6 year). Ninety percent of pregnancies had normal chromosomes and 10% of the cases had numerical chromosomal abnormalities. Trisomy 21 was the most frequent chromosomal disorder across all indications (5%), followed by Turner syndrome (2%), trisomy 18 (2%), and trisomy 13 (1%). When comparing the FISH data with karyotype results for chromosomes 13, 18, 21, X, and Y in the 83 individual tested, no false positive or negative results were detected by the FISH assay. The result obtained by FISH and the banding cytogenetic were in complete accordance.

**Conclusion:**

This study supports the integration of amniotic fluid (AF) FISH as a RAS test, in to routine antenatal practice for identification of chromosome aneuploidies. There are trends towards delayed childbearing and most cases of Down Syndrome (DS) are currently detected post-nataly in the Egyptian population. Consequently, the live birth prevalence of DS has increased, which might lead to a serious negative public health effects.

## Introduction

Over the last 30 years, U.S. Agency for International Development (USAID) and Egypt’s primary focus was on assisting the Ministry of Health and Population (MOHP) to increase the availability of family planning (FP) and public health services. The Egyptian FP and reproductive health (RH) program has achieved considerable success over the last three decades, preventing millions of infants and child deaths and high risk-births in Egypt and saving tens of thousands of mothers’ lives. Before the program began in the 1970 the country had hardly any FP services or products, maternal and infant mortality rates were high, and population growth was straining the country resources (Adel-Tawab et al. 2008).

Impressive improvements in maternal health care are saving women’s life throughout Egypt. The changes were accomplished in less than 8 years (1993–2000) by employing a systemic approach to addressing avoidable causes of death. An essential package of Maternal and Child Health (MCH) services and standards of antenatal care (ANC) and postnatal care, delivery and obstetric care, neonatal care, and preventive child death was applied in targeted governorates reducing suffering and increasing lives saved. Egypt is now on track to achieve Millennium development Goal (MDG) 5 of improving maternal health. The target aims at reducing maternal mortality rate (MMR) by three-quarters between 1990 and 2015 (Cobb et al. 1993).

The policy of MOHP for antenatal care currently calls for, monitoring the progress of pregnancy and regularly assessing maternal and fetus well-being routinely for all pregnant women. At least four visits are recommended and should cover detection of problems complicating pregnancy (e.g., anemia, hypertensive disorders, bleeding, mal-presentations, and multiple pregnancies); preventive health care interventions, such as tetanus toxoid immunization, and iron, folate and iodine supplements (Chao et al. 2005).

Although there has been an improvement in ANC in Egypt, there has not been enough focus on antenatal screening to pregnant women for fetuses with chromosomal disorders like DS and other rare disorders like trisomy 18 and neural tube defects. Prenatal screening is still inaccessible to most families and almost all cases of DS are diagnosed post-natal (El-Sobky and Elsayed 2004).

Prenatal diagnosis for DS has made considerable progress in the past twenty years. In particular, techniques using maternal serum and ultrasonography markers have provided non-invasive antenatal screening tests for DS. This has been especially important for younger women who are at lower risk of DS, and hence usually not candidates for invasive diagnostic procedures, but who nevertheless often account for the majority of DS cases due to the size of their population (Wald et al. 1999;Nicolaides et al. 2002).

Along with technical progress in antenatal screening, public policies on screening have been adopted in several European countries. The majority of countries have moved from solely offering older mothers a diagnostic test (chorionic villous sampling or amniocentesis) to having some form of DS screening in place, with over half having country-wide policy or recommendation for first or second trimester screening (Wellesley et al. 2002).

The policy and practice of prenatal screening for chromosomal anomalies is changing rapidly. New developments in screening methods have increased the number of options for patients. Screening options in the first trimester include nuchal translucency (NT) testing in combination with measurement of pregnancy-associated plasma protein A (PAPP-A) and human chorionic gonadotropin (hCG). Screening options in the second trimester include serum screening using triple (hCG, maternal serum alpha fetoprotein (AFP), unconjugated estradiol) or quadruple screening (hCG, inhibin A, maternal serum AFP, unconjugated estradiol), and ultrasonography (Boyd et al. 2008).

Having a screening policy in place had a measurable impact on prenatal detection rates for DS, the registry areas in countries offering primarily first-trimester screening had significantly higher detection rate than those using a mixed first or second trimester screening policy; those with some screening but no national policy in place were significantly less likely to detect a DS case prenatally (Anderson and Brown 2009).

After a positive prenatal screening test, women are usually offered foetal karyotyping, which is considered the gold standard to confirm the presence or absence of chromosomal abnormality by counting the number of chromosomes and looking for structural abnormality (Gekas et al. 2011).

Karyotyping involves the acquisition of metaphase chromosomes through a period of cell culture that may take anywhere between 7 and 14 days. Despite increasingly shorter prenatal result turn-around times due to the wider adoption of the in-situ coverslip technique and improved culture media, patients remain anxious while waiting for the1 to 2 weeks it takes for an AF or chorionic villous sample (CVS) karyotype result (Lim et al. 2010).

The most frequent foetal chromosomal abnormalities involve the autosomes 21, 18, 13, and sex chromosomes X and Y. Aneuploidy or alterations in copy number of these chromosomes, including trisomy 21 (Down syndrome), trisomy 18 (Edwards’ syndrome), trisomy 13 (Patau’s syndrome), 45, X (Turner’s syndrome), and 47, XXY (Klinefelter’s syndrome) account for 80% of clinically significant chromosomal abnormalities diagnosed in the prenatal period (Leung et al. 2008).

Consequently, RAS tests such as (FISH) and quantitative fluorescence-polymerase chain reaction (QF-PCR) assays to detect numerical abnormalities of chromosomes 13, 18, 21, X and Y have become increasingly popular adjunct tests. Unlike karyotyping, these test results are typically available within 24 hours thereby alleviating much of the anxiety from these patients. This has been one of the main reasons for the introduction of molecular (cytogenetic) methods for prenatal diagnosis of the most common chromosome disorders (Liehr and Ziegler 2005).

Currently, there is no national prenatal screening program in Egypt; some pregnant mothers obtain screening as a part of their obstetric care in the private sector on a fee –for- service basis.

The objective of this study is to evaluate the usefulness and limitations of FISH as a tool in RAS for high risk aneuploidies (13, 21, 18, X, Y) before its approval in the national antenatal screening and genetic diagnosis program in Egypt.

## Patients and methods

### Patients

From January 2009 to December 2009, 437 pregnant women were referred to the MGC for prenatal diagnosis. Our policy is to provide all women with interphase FISH assay within 48 hours followed by cytogenetic analysis. If rapid analysis by FISH was considered as a replacement for standard karyotyping, the indication for prenatal diagnosis should be either positive screening test for DS or advanced maternal age (AMA). During counseling, different methods of prenatal diagnosis are discussed, and the final decision is left to the parents.

In this study, interphase FISH analysis and the cytogenetic data and of 100 consecutive patients who agreed to have FISH and cytogenetic analysis in the study period were retrieved and reviewed. Seventeen patients had FISH analysis only due to insufficient sampling.

The clinical indications of prenatal diagnosis in this study included, abnormal maternal serum screening (MSS) if the risk reached or exceeded 1 in 300 for trisomy 21 or 1 in 150 for trisomy 13/ trisomy 18, abnormal ultrasound (AU), AMA (≥35 years), family history of genetic/chromosomal disorders (previous DS), and prenatal anxiety. Amniocentesis was performed at around 16 weeks of gestation and usually 20 ml of fluid was collected. CVS samples were performed at 12 weeks of gestation trans-abdominally and villi were processed for direct harvesting, culture and FISH.

### Fluorescence in-situ hybridization

#### Fluorescence in-situ hybridization probes

The FISH assay employed the AneuVysion Assay Kit (Abbot Molecular, US) consisting of three α-satellite DNA probes for chromosomes X, Y, and 18 (cep X, cep Y, cep 18) and two locus specific probes (LSI) for 13q14 (LSI 13) and 21q22.13-22.2 (LSI 21). The three centromeric probes and the two locus-specific probes are applied to the sample in two different hybridizations slides.

### Cytogenetic analysis

Amniocytes were cultured and Q banding was performed for most of the cases. Routine evaluation of each case involved analysis of 20 random metaphase spread from two independent cultures. Four metaphase spreads were photographed for karyotyping using imaging system. When mosaicism was suspected more than 50 metaphase spreads were analyzed. Karyotypes were described according to the International System for Human Cytogenetic Nomenclature (2005) (ISCN2005).

### Preparation of uncultured amniocytes

About 2 to 5 ml of AF were centrifuged (1000 rpm, 5 min) and the cell pellet was re-suspended in 3 ml trypsin/EDTA and incubated for 15 min at 37°C. After centrifugation, the pellet was suspended in 5 ml 0.075 KCL and incubated at 37°C for 20 min. After addition of 2 ml Carnoy fixative [methanol: acetic acid (3:1)], centrifugation, re-suspension, and incubation at -20°C for 5 min in 3 ml carnoy fixative. The supernatant was discarded and the cells were diluted in 200 μl of the remaining supernatant. The cells of each sample were smeared on two different slides, one slide for hybridization with probes LSI 13 (green) and 21 (red), and the other slide for hybridization with probes CEP 18 (blue), CEP X (green), and CEP Y (red).

For hybridization, the slides were incubated with pepsin in 0.01 mol/L HCL at 37°C for 8 min. Then the slides were washed with 2× SCC for 2 min, and dehydrated with ethanol at 70%, 85%, and 100% in sequence, and air-dried. Each probe mixture was individually dropped on one slide, covered by a cover glass and sealed with rubber cement. Co-denaturation and hybridization were carried out in a HYBrite system (Abbott) overnight. The next day, the slides were washed and prepared for analysis.

### Analytical criteria

For each probe, at least fifty nuclei were evaluated. Nuclei free from any attached cytoplasm or cellular membrane showing 1, 2, 3, or 4 signals were selected for scoring. Only those signals, which were well embedded in the nucleus, were included for scoring. Clumped or overlapping nuclei and nuclei with high background intensity or low signal intensity were not scored. Patchy and diffused signals were included in the evaluation only if they were well separated. Split-spots (i.e. signals in a paired arrangement) were scored as one signal only if the distance between the signals was less than the width of one of these signals; otherwise they were observed as two signals. On the basis of FISH scoring results, the samples were considered to be informative normal and abnormal (however final diagnosis was made only on the basis of karyotyping). Informative normal/disomic samples were defined as samples in which ≥ 80% of all nuclei from each autosomal hybridizations demonstrated two signals and ≥ 80% of all nuclei from the sex chromosomal hybridization demonstrated the XX or XY signal pattern. Informative abnormal specimen were defined as those in which ≥ 70% of the nuclei spreads hybridized with an autosomal probe demonstrated 3 signals or ≥ 70% of the nuclei spreads hybridized with the X- and Y-probes demonstrated signal patterns other than XX- and XY-signals.

As long as not more than 7-10% of the studied cells were present with three specific signals, no trisomy of the corresponding chromosomal region is suspected and a normal report (disomic) is issued.

Informative mosaic samples were defined as those in which a value of > 20% of nuclei had a variation in signal number from the majority or showed a signal pattern other than the normal disomic autosomal or normal sex chromosomal (XX and XY) signals. At between 7-10% and 20%, the evaluation was regarded as unclear result and the number of cells evaluated was increased to 100 or more nuclei to come to a final decision. If the above criteria were not met the result were reported as uninformative (Liehr and Ziegler 2005;Jobanputra et al. 2002).

Owing to the fact that in amniotic fluid of male fetuses, maternal cell contamination can be detected but in fluid of female fetuses, such contamination could not be detected. Therefore, the results for normal female fetuses were interpreted carefully using lower cut-off values.

## Results

A total number of 100 consecutive prenatal samples were analyzed among, 437 amniocentesis in the MGC from January 2009 to December 2009. Maternal age varied from 21 to 44 years (mean was 35.6 year) and gestational age at the time of the procedure varied from 12 to 27 weeks (mean age was 16 + 4 weeks).

From a total number of 100 pregnant women, 17 had only prenatal FISH assay and 83 had conventional karyotype and prenatal FISH analysis. The FISH analysis showed that 90% of pregnancies had normal chromosomes and 10% of the cases had numerical chromosomal abnormalities.

Chromosome anomalies identified by the FISH technique were 8 cases (8%) of autosomal aneuploidies and two cases (2%) of gonosomal aneuploidies. Trisomy 21 was the most frequent chromosomal disorder across all indications (5%), followed by Turner syndrome (2%), trisomy 18 (2%), and trisomy 13 (1%).

When comparing the FISH data with karyotype results for chromosomes 13, 18, 21, X, and Y in the 83 individual tested, no false positive or negative results were detected by the FISH assay. The result obtained by rapid aneuploidy test and the banding cytogenetic were in complete accordance.

The clinical indications for prenatal genetic diagnosis included, abnormal MSS (50%), AU findings (17%), (AMA) (16%), family history of genetic/chromosomal disorder (previous DS) (8%), and prenatal anxiety (9%). In cases where there were multiple indications, priority of indications was assigned as follows: AU, MSS, and AMA. The majority of abnormalities identified by prenatal studies were due to MSS (4%), UA (4%), and AMA (2%), Table 1.

**Table 1:** **The analysis of the recommendations for amniocentesis that conducted to the discovery of the numerical chromosome abnormalities**

Indications	Number of patients	Trisomy 21	Trisomy 18	Trisomy 13	Monosomy X	Total aneuploidy N (%)
Maternal serum screening positive	50	2	1	0	1	4/50 (8)
Abnormal ultrasoungraphic findings (US)	17	1	1	1	1	4/17 (23.5)
Advanced maternal age (≥ 35 years)	16	2	0	0	0	2/16 (12.5)
Family history of genetic/chromosomal disorder	8	0	0	0	0	0
Parental anxiety	9	0		0	0	0
Total	100	5	2	1	2	10

All FISH studies were completed and reported within 24–72 hours, while the time varied from 2–3 weeks for conventional karyotyping. FISH results were reported to be significantly faster than those for karyotyping. However, 5% of cases were problematic due to inadequate cell number, maternal cell contamination or hybridization failure. In these cases an adequate FISH result was obtained by repeating FISH on the same slide or analyzing floating amnion cells from supernatant in cell culture flaks.

## Discussion

Amniocentesis is the most common invasive prenatal procedure for detection of fetal chromosomal abnormalities.

In this study, abnormal MSS was the most common indication. This finding was similar to the results of previous studies (Choi et al. 2005;Jang et al. 2007;Han et al. 2008). In contrast, recent studies performed in Egypt showed that previous history of an abnormal child was the most common indication for amniocentesis (Helmy et al. 2009;Atef et al. 2011). This difference reflects the increased awareness of our patients about the importance of prenatal serum screening tests. It should be considered that MGC is a private laboratory, and most of our patients come from high social class families, when compared to the previous studies performed at governmental hospitals where free or minimum charge prenatal diagnosis is provided for high risk group only.

Previous reports on prenatal diagnosis of amniocentesis, consisting of various numbers of cases, have revealed that the incidence of chromosomal abnormalities ranges from 1% and 6.7% (Tseng et al. 2006;Han et al. 2008which was similar to the data from Egyptian study by Shawky et al. 2009.

In this study, DS was the most common abnormality detected (5%) by prenatal diagnosis and at the same year 2009, 429 (8.7%) live- birth DS were diagnosed postnatally in the MGC. Prenatal diagnosis of DS is not available for most of pregnant women in Egypt and the National DS cytogenetic register is not yet developed to compare our results. However, there are several reports on the increased incidence of DS from different parts of the world, with respect to ethnicity and maternal age (Warburton et al. 2004;Lamb et al. 2005). An earlier study in Egypt have reported the incidence of DS as, 1 per 700 births and 98.4% of cases were diagnosed postnatal and only 1.56% were detected prenatally with an estimated risk of 2285 DS births annually (El-Sobky and Elsayed 2004). Our result suggests an increased awareness of our group of patients about the importance of antenatal screening for DS. On the other hand, the MOHP in Egypt did not adopt proper antenatal screening as a public policy till today (El-Gilany et al. 2011;Shalaby 2011).

There are two factors known to influence the number of live births with DS- the underlying incidence of the syndrome to the distribution of maternal age, and the number of pregnancies that are detected antenatal and subsequently terminated (Shalaby 2011;Morris and Alberman 2009). In this work, the mean age of mothers was advanced (35.6 years) when compared to the mean maternal age in Egypt (21.9 ± 3.45 years, 2008), (Finaly et al. 2011).

Traditionally, screening for trisomy 21 was based on maternal age and biochemical testing of second trimester (14–18 weeks) maternal serum AFP (Snijders et al. 1998). However, it can now be provided effectively by a combination of the ultrasonography measurement of fetal NT thickness, absence/presence of nasal bone (NB) in conjunction with biochemical testing for PAPP-A and hCG at 11 weeks to 13 weeks and six days (13 + 6 weeks) of gestation. The detection rate of these combined methods is about 85-90% in regard to trisomy 21 and 18, for a false positive rate of 5% (Agnieszka et al. 2007).

In previous studies (El-Sobky 2007;Tseng et al. 2006;Yang et al. 1999), the AU findings showed the highest detection rate for chromosomal abnormalities in prenatal diagnosis. In the present study, of the 17 cases with AU findings, 4 cases resulted in chromosomal abnormalities, which showed the highest positive predictive value (23%) among the indications. Nowadays, highly sensitive ultrasonic technology can detect fetal anomalies which eventually necessitate amniocentesis.

In this study, (90%) of amniocentesis performed for patients showed normal result. For this large group of parents, RAS using FISH excluded the possibility of foetal DS and relieved anxiety within 1–2 days. However, the final and conclusive result was not available until a full karyotype was obtained in the week following the FISH finding, in which abnormalities not detectable by FISH was unveiled.

Currently, most prenatal diagnosis units offer either traditional karyotyping only or RAS in addition to karyotyping. If cost is not an issue, the latter appears ideal. However in publicly funded system, money spent in one area means deprivation in another.

There is an ongoing debate surrounding whether RAS should be employed as an adjunct to karyotyping or whether it could be used as a stand-alone test in selected group of women (Leung et al. 2003;Caine 2005;Leung 2005;Bui 2007). The controversy is due to the residual probability of a chromosome abnormality (both balanced and unbalanced) when RAS demonstrates a normal result.

In this work, the result obtained by RAS for common aneuploidies and the banding cytogenetic were in complete accordance with no false positive or false negative results. Similar results were obtained by (Pergamnet et al. 2000;Lim et al. 2002;Settin et al. 2007;Neagos et al. 2011). The relatively small number of cells needed for diagnosis (practically 50), gave a much better option to be more selective in cell quality and was a major factor in the efficiency and accuracy of the tests.

Although RAS using FISH is highly sensitive and specific method in the detection of aneuploidy, one of the disadvantages of FISH is that maternal and fetal XX cells *per se* are indistinguishable by FISH, rendering maternal cell contamination undetectable from female fetuses. However, maternal cell contamination is readily detectable with male fetuses, as a mixture for XX and XY cells are seen. In contrast to the situation of FISH, maternal cell contamination is readily detected by careful comparison with profiles from maternal blood sample in QF-PCR amplification of STR (short tandem repeats) (Bui 2007;Hulten et al. 2003). For safety, we and many laboratories discard any heavily contaminated samples with respect to RAS.

In contrast to the RAS, the array-based comparative genomic hybridization (a CGH) is a comprehensive, high resolution, genome-wide screening strategy for obtaining DNA copy number information in a single measurement which can be rapid and less laborious than karyotyping as it is readily amenable to automation. While currently high costs of the technology need to be addressed before it can be used in prenatal diagnosis, it is likely to become an important tool with the potential of replacing karyotyping in the future (Bui 2007).

The feasibility of the RAS stand-alone approach depends on the indication for the invasive prenatal test. With the presence of major ultrasound-detected foetal anomalies, traditional karyotyping should be performed to look for structural chromosomal abnormalities apart from aneuploidies. The RAS stand-alone approach is best when the invasive prenatal test is performed for an identified increased risk of Down syndrome from a positive screening test (Leung et al. 2008).

For most women, when the indication for prenatal diagnosis is AMA (in isolation or combined with maternal serum and ultrasonographic screening for fetal (NT), this risk is usually relatively low, in order of 0.1-0.2% (Grimshaw et al. 2007). In other words, in the low risk group of women, the abnormality detection rate is around 99.9-99.8%. In contrast, once a structural abnormality of the fetus has been diagnosed using ultrasonography, the risk may be substantially increased. The risk of fetal chromosome abnormality is increased when either parent is a carrier of a chromosome rearrangement such as translocation, inversion, or insertion. Neither FISH nor QF-PCR aneuploidy assay are applicable. Either karyotyping or specific molecular testing is mandatory (Chen et al. 2001).

Our findings show that all clinically significant numeric cytogenetic abnormalities (trisomy 21, trisomy 13, and trisomy 18, and sex chromosome abnormalities) can be detected if RAS was used as a stand-alone approach. On the other hand, to carry out RAS FISH, amniocentesis or CVS still needs to be performed. These invasive techniques carry an intrinsic risk of miscarriage of 0.5% and 1% respectively. With small as such risk may be, it would seem prudent to have a thorough karyotype analysis to reveal the maximum information possible (Gekas et al. 2011).

While the use of RAS to give a rapid result for the common aneuploidies as adjunct to karyotyping is recommended for routine prenatal diagnosis, this combined approach clearly increases the cost of prenatal diagnosis. However, the quality of life and anxiety measurements show a significantly increased health status after diagnosis with RAS, and RAS allows earlier decision making in cases where the fetus has a detected chromosomal abnormality (Gekas et al. 2011).

In addition, a joint statement by the American College of Medical genetics and the American society for Human genetics reaffirmed that all RAS tests results must be followed up with karyotyping reports indicated that 15-30% of cytogenetic abnormality detected by karyotyping would not be detected by RAS, but the number of these cytogenetic abnormality with risk of adverse outcome above background levels are much lower, and the relevance of diagnosing them via DS screening programs is debated owing their clinical significance (Lim et al. 2010).

On the other hand, the termination of pregnancy remains a contentious issue in many societies, despite the socially acceptable view of abortion as immoral; many Muslims would personally accept prenatal testing technology and may opt for termination of pregnancy for conditions perceived to be burdensome for the child (Ahmad et al. 2013). While this may be acceptable to literate Muslims, much of the Muslim population depend on the local Imams (Muslim leaders), most of whom are strongly against termination of life (Salihu 1997).

The Egypt population has reached 91 million by the end of August 2012, and with the increase in the average growth rate to reach 2, we expect to have an added 2850 DS birth every year. Considering suffering family members, it means that an average of 11,400 family member suffering both social and psychological, especially with the very limited resources for handicapped children.

In economically developed countries, life expectancy for individuals with DS continues to rise, and significant, if uneven, progress has been made in education provision and employment prospects for those with an intellectual disability (Glasson et al. 2002). In contrast, the limited literature from economically developing countries tends to characterize life for people with intellectual disability and their families as burdensome and stigmatized (Ghai 2001). A large number of individuals cannot afford medical insurance and make use of the free-of-charge state sector medical services; nonetheless, the utilization of these services is impeded by transport costs, bureaucracy and complex management patterns. Therefore, the only public health measure available to reduce the incidence of DS births is antenatal screening followed by invasive prenatal genetic diagnosis and selective therapeutic abortion of affected fetuses (Scott et al. 2013).

## Conclusion

We demonstrate that AF FISH as a RAS test, can provide a rapid and accurate clinical method for prenatal identification of chromosome aneuploidies as a stand-alone test or adjunctive to karyotyping according to the indication for amniocentesis. Due to the development of maternal serum markers and sensitivity of ultrasonic technology, the indications for amniocentesis are changing in Egypt in the high social class sector of the Egyptian population. The proportion of pregnancies with advanced maternal age has increased, and in the absence of proper antenatal screening and subsequent termination it will lead to an increase in the incidence of live births with DS. All pregnant women should have proper ANC which includes prenatal screening for chromosomal aneuploidies as a part of the Egyptian National Health Service.

## Abbreviations

RAS: Rapid aneuploidy screening
MGS: Medical genetics center
DS: Down syndrome
USAID: U.S. agency for international development
MOHP: Ministry of health and population
FP: Family planning
RH: Reproductive health
MCH: Maternal and child health
ANC: Antenatal care
MDG: Millennium development Goal
MMR: Maternal mortality rate
FISH: Fluorescent in situ hybridization
QF-PCR: Quantitative fluorescence-polymerase chain reaction
MSS: Maternal serum screening
AU: Abnormal ultrasound
AMA: Advanced maternal age
CVS: Chorionic villous sample
AF: Amniotic fluid
NT: Nuchal translucency
PAPP-A: Pregnancy associated plasma protein A
hCG: Human chorionic gonadotropin
AFP: Alpha fetoprotein
NB: Nasal bone.
